# Reoccurrence of Avian Influenza A(H5N2) Virus Clade 2.3.4.4 in Wild Birds, Alaska, USA, 2016

**DOI:** 10.3201/eid2302.161616

**Published:** 2017-02

**Authors:** Dong-Hun Lee, Mia K. Torchetti, Mary Lea Killian, Thomas J. DeLiberto, David E. Swayne

**Affiliations:** US Department of Agriculture, Athens, Georgia, USA (D.-H. Lee, D.E. Swayne);; US Department of Agriculture, Ames, Iowa, USA (M.K. Torchetti, M.L. Killian);; US Department of Agriculture, Fort Collins, Colorado, USA (T.J. DeLiberto)

**Keywords:** highly pathogenic avian influenza virus, influenza A(H5N2) virus, viruses, influenza, H5N2 subtype, waterfowl, wild birds, phylogenetic analysis, clade 2.3.4.4, zoonoses, North America, Alaska, United States

## Abstract

We report reoccurrence of highly pathogenic avian influenza A(H5N2) virus clade 2.3.4.4 in a wild mallard in Alaska, USA, in August 2016. Identification of this virus in a migratory species confirms low-frequency persistence in North America and the potential for re-dissemination of the virus during the 2016 fall migration.

Historically, apparently effective geographic barriers (Bering and Chukchi Seas of the North Pacific Ocean) appeared to limit dissemination of Asian-origin, highly pathogenic avian influenza virus (HPAIV), such as influenza A(H5N1) virus A/goose/Guangdong/1/1996 (Gs/GD), between the Old and New Worlds (*1*). However, such barriers are incomplete; occasional spillovers of virus genes move from 1 gene pool to another (*2*). Asian-origin HPAIV H5N8 was identified in North America at the end of 2014 (*3*).

Novel HPAIVs H5N1, H5N2, and H5N8 emerged in late 2014 by reassortment with North American low pathogenicity avian influenza viruses (*4*). A novel reassortant H5N2 virus originating from Asian-origin H5N8 virus clade 2.3.4.4 and containing Eurasian polymerase basic 2, polymerase acidic, hemagglutinin, matrix, and nonstructural protein genes and North American lineage neuraminidase (NA), polymerase basic 1 (PB1), and nucleoprotein genes was identified on poultry farms in British Columbia, Canada, and in wild waterfowl in the northwestern United States. This virus subsequently predominated during influenza outbreaks in the United States in 2015.

During the boreal summer, birds from 6 continents (North America, South America, Asia, Africa, Australia, and Antarctica) fly to Alaska, USA, to breed. Thus, Alaska is a potentially major location for intercontinental virus transmission (*1*,*2*). Recent data provide direct evidence for viral dispersal through Beringia (*5*,*6*). Genetic evidence and waterfowl migratory patterns support the hypothesis that H5 virus clade 2.3.4.4 was introduced into North America through the Beringian Crucible by intercontinental associations with waterfowl (*3*). In addition, low pathogenicity avian influenza viruses were collected in Alaska before initial detection of H5 HPAIV clade 2.3.4.4, which contained genes that had recent common ancestry with reassortant H5N2 virus PB1, nucleoprotein, and NA (N2 subtype) genes and H5N1 virus PB1, polymerase acidic, NA (N1 subtype), and nonstructural protein genes of HPAIVs (*7*).

We report detection of an HPAIV H5N2 subtype from wild mallard sampled in Alaska during August 2016. Influenza A virus was detected in 48/188 dabbling duck samples collected during a live bird banding effort near Fairbanks, Alaska, during August 6–15, 2016. One sample of H5 virus from an adult mallard was identified as an HPAIV H5N2 on the basis of complete genome sequencing. We conducted comparative phylogenetic analysis of A/mallard/Alaska/AH0008887/2016(H5N2) virus, hereafter known as 8887/2016(H5N2) virus, to trace its origin and understand its genetic relationship to HPAIV H5N2 isolated in 2014–2015 (Technical Appendix).

We considered 8887/2016(H5N2) virus an HPAIV on the basis of amino acid sequence at the hemagglutinin proteolytic cleavage site (PLRERRRKR/G), as shown for other Gs/GD HPAIV H5Nx subtypes in subclade 2.3.4 (http://www.offlu.net/fileadmin/home/en/resource-centre/pdf/Influenza_A_Cleavage_Sites.pdf). Homology BLAST searches showed that all genes had >99.2% nucleotide similarity with genes of H5N2 virus outbreak strains collected during late February–March 2015 (Technical Appendix Table).

Phylogenetic analysis showed that the concatenated genome of 8887/2016(H5N2) virus formed a cluster with viruses from initial detections in the midwestern United States, including a snow goose in Missouri, a backyard poultry farm in Kansas, and a turkey farm in Minnesota (Figure). Our epidemiologic investigation data suggested that point-source introductions by indirect contact with wild waterfowl were the most probable source of infection for these backyard poultry in Kansas and a turkey farm in Minnesota (*8*). This genetic cluster was supported by a maximum-likelihood bootstrap value of 80 and a Bayesian posterior probability of 1.00.

**Figure:**
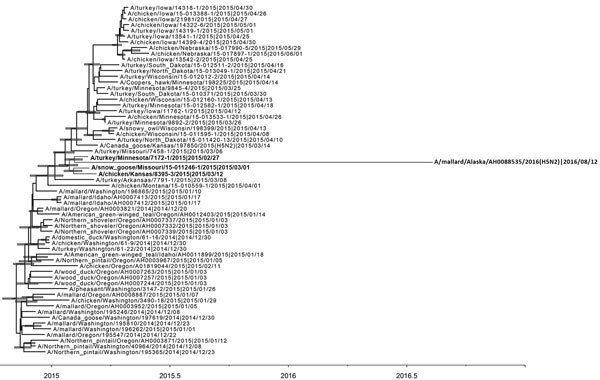
Maximum clade credibility phylogeny of concatenated complete genome sequences of avian influenza A(H5N2) virus clade 2.3.4.4 in wild birds, Alaska, USA, 2016. Horizontal bars indicate 95% Bayesian credible intervals for estimates of common ancestry. Bold indicates a genetic cluster that includes A/mallard/Alaska/AH00088535/2016/08/12(H5N2) virus and related viruses. Scale bar indicates years.

The mean time to most recent common ancestry of viruses in this genetic cluster was estimated to be the end of January 2015 (mean time to most recent common ancestry January 27, 2015, 95% Bayesian credible interval January 11–February 10, 2015). Consistent clustering of 8887/2016(H5N2) virus with other H5N2 outbreak viruses in phylogenies for each gene suggests that the 8887/2016(H5N2) virus probably evolved through genetic drift from common ancestors of outbreak viruses in the absence of further reassortment (Technical Appendix Figure 2). The mean rate of the nucleotide substitution obtained by Bayesian analysis was 6.064 × 10^–3^ (95% Bayesian credible interval 4.43–7.82 × 10^–3^) substitutions/site/year. In the root-to-tip regression plot of maximum-likelihood phylogeny, we found that 8887/2016(H5N2) virus fell below the regression line, which indicated sequences that are slightly less divergent than average of 2014–2015 H5N2 outbreak viruses (Technical Appendix Figure 3).

The last reported detection during the influenza outbreak in the United States in 2015 was from a Canada goose in Michigan on June 17. There were 2 detections by PCR (3 assays, 2 gene targets, no virus recovered, no sequence obtained) from mallards in July (bird banding effort in Utah) and November (hunter harvest in Oregon) during surveillance in 2015–2016. Sequence of the HPAIV H5N2 from a wild mallard during surveillance in 2016–2017, evidence for continued evolution of this virus lineage, widespread detections of HPAIV H5N2 in healthy wild birds (*9*), and lack of pathobiological effects in experimentally infected waterfowl (*10*) collectively provide strong evidence for maintenance of HPAIV H5N2 in wild birds in North America. Detection of HPAIV in a mallard might imply the potential for dissemination of HPAIV H5N2 during the southward fall migration of waterfowl in 2016.

Technical Appendix Additional information on reoccurrence of avian influenza A(H5N2) virus clade 2.3.4.4 in wild birds, Alaska, USA, 2016.
