# Supplementary material for: Reoccurrence of Avian Influenza A(H5N2) Virus Clade 2.3.4.4 in Wild Birds, Alaska, USA, 2016
Source: Emerg Infect Dis. 2017 Feb;23(2):365–7. doi: 10.3201/eid2302.161616 (PMC5324823; doi:10.3201/eid2302.161616)
Supplement: Technical Appendix — Additional information on reoccurrence of avian influenza A(H5N2) virus clade 2.3.4.4 in wild birds, Alaska, USA, 2016. [file 16-1616-Techapp-s1.pdf]

Article DOI: http://dx.doi.org/10.3201/eid2302.161616

Reoccurrence of Avian Influenza A(H5N2) Virus Clade 2.3.4.4 in Wild Birds, Alaska, USA, 2016

Technical Appendix

Methods for Genome Sequencing and Phylogenetic Analysis

We collected 188 wild waterfowl samples from Creamer's Field Migratory Waterfowl Refuge located in Fairbanks, Alaska, USA during August 6–15, 2016. We conducted complete genome sequencing and comparative phylogenetic analysis of A/mallard/Alaska/AH0008887/2016(H5N2) virus, hereafter 8887/2016(H5N2), to trace the origin and to estimate its evolutionary history. A sample was confirmed to be H5 positive by using matrix gene real-time reverse transcription PCR and genome sequencing.

Complete genome sequencing of 8887/2016(H5N2) virus was performed by using next-generation sequencing with Ion Chef, the Ion S5 sequencing system, and Ion Total RNA-Seq Kit v2 Library Preparation Kit (Thermo Scientific Fisher, Waltham, MA, USA) according to the manufacturer`s instructions. Data were analyzed by sing SeqMan NGen v. 4 (https://www.dnastar.com/t-nextgen-seqman-ngen.aspx). Nucleotide sequences were deposited in GenBank under accession nos. KX838896–KX838903.

For phylogenetic analysis, we retrieved and used all H5N2 highly pathogenic avian influenza virus subtype sequences identified in North America during 2014–2015 available in the Influenza Virus Resource (https://www.ncbi.nlm.nih.gov/genome/viruses/variation/flu/) as of September 1, 2016. Maximum-likelihood (ML) phylogenies of each gene segment and concatenated full genome were generated by using RAxML (*1*) and the Generalized Time Reversible nucleotide substitution model with among-site rate variation modeled by using a discrete gamma distribution.

ML phylogenies of polymerase basic 2, polymerase acidic, hemagglutinin, matrix, and nonstructural protein genes were rooted to A/Crane/Kagoshima/KU1/2014(H5N8) virus, and polymerase basic 1, nucleoprotein, and neuraminidase genes were rooted to low pathogenicity avian influenza viruses collected in Alaska that share recent common ancestry with H5N2 subtype to highly pathogenic avian influenza viruses (*2*). Bootstrap support values were generated by using 1,000 rapid

bootstrap replicates. To investigate the temporal signal and clocklikeness of ML phylogenies of the dataset, we performed linear regression on the root-to-tip distances of samples versus date of the isolate by using TempEst v1.5 (*3*).

Bayesian relaxed clock phylogenetic analysis of concatenated genome (ntax = 61) was performed by using BEAST v1.8.3 (*4*). We applied an uncorrelated lognormal distribution relaxed clock method, the Hasegawa–Kishino–Yano nucleotide substitution model and the Bayesian skyline coalescent prior. A Markov Chain Monte Carlo method to sample trees and evolutionary parameters was run for 1.0×10^8 generations. At least 3 independent chains were combined to ensure adequate sampling of the posterior distribution of trees. BEAST output was analyzed with TRACER v1.4 (https://beast.bio.ed.ac.uk/tracer) with 10% burn-in. A maximum clade credibility tree was generated for each dataset by using TreeAnnotator in BEAST. FigTree 1.4.2 (https://tree.bio.ed.ac.uk/) was used for visualization of trees.

References

1. Stamatakis A. RAxML version 8: a tool for phylogenetic analysis and post-analysis of large phylogenies. Bioinformatics. 2014;30:1312–3. PubMed http://dx.doi.org/10.1093/bioinformatics/btu033

2. Ramey AM, Reeves AB, TeSlaa JL, Nashold S, Donnelly T, Bahl J, et al. Evidence for common ancestry among viruses isolated from wild birds in Beringia and highly pathogenic intercontinental reassortant H5N1 and H5N2 influenza A viruses. Infect Genet Evol. 2016;40:176–85. PubMed http://dx.doi.org/10.1016/j.meegid.2016.02.035

3. Rambaut A, Lam TT, Max Carvalho L, Pybus OG. Exploring the temporal structure of heterochronous sequences using TempEst (formerly Path-O-Gen). Virus Evol. 2016;2:1ew007. eCollection 16.

4. Drummond AJ, Rambaut A. BEAST: Bayesian evolutionary analysis by sampling trees. BMC Evol Biol. 2007;7:214. PubMed http://dx.doi.org/10.1186/1471-2148-7-214

Technical Appendix Table. Nucleotide identities between A/mallard/Alaska/AH0008887/2016(H5N2) influenza virus and nearest homologs in GenBank as of September 1, 2016

Gene*	Virus	Collection date, 2015	% Identity
PB2	A/Canada goose/Kansas/197850/2015(H5N2)	Mar 13	99.7
PB1	A/turkey/Minnesota/7172–1/2015(H5N2)	Feb 27	99.6
PA	A/turkey/Minnesota/7172–1/2015(H5N2)	Feb 27	99.7
HA	A/snow goose/Missouri/15–011246–1/2015(H5N2)	Jan 3	99.4
NP	A/turkey/Minnesota/7172–1/2015(H5N2)	Feb 27	99.5
NA	A/turkey/Missouri/7458–1/2015(H5N2)	Mar 6	99.5
MP	A/Canada goose/Kansas/197850/2015(H5N2)	Mar 13	99.6
NS	A/snow goose/Missouri/15–011246–1/2015(H5N2)	Jan 3	99.2

*HA, hemagglutinin; MP, matrix; NA, neuraminidase; NP, nucleoprotein; NS, nonstructural; PA, polymerase acidic; PB1 polymerase basic 1; PB2, polymerase basic 2.

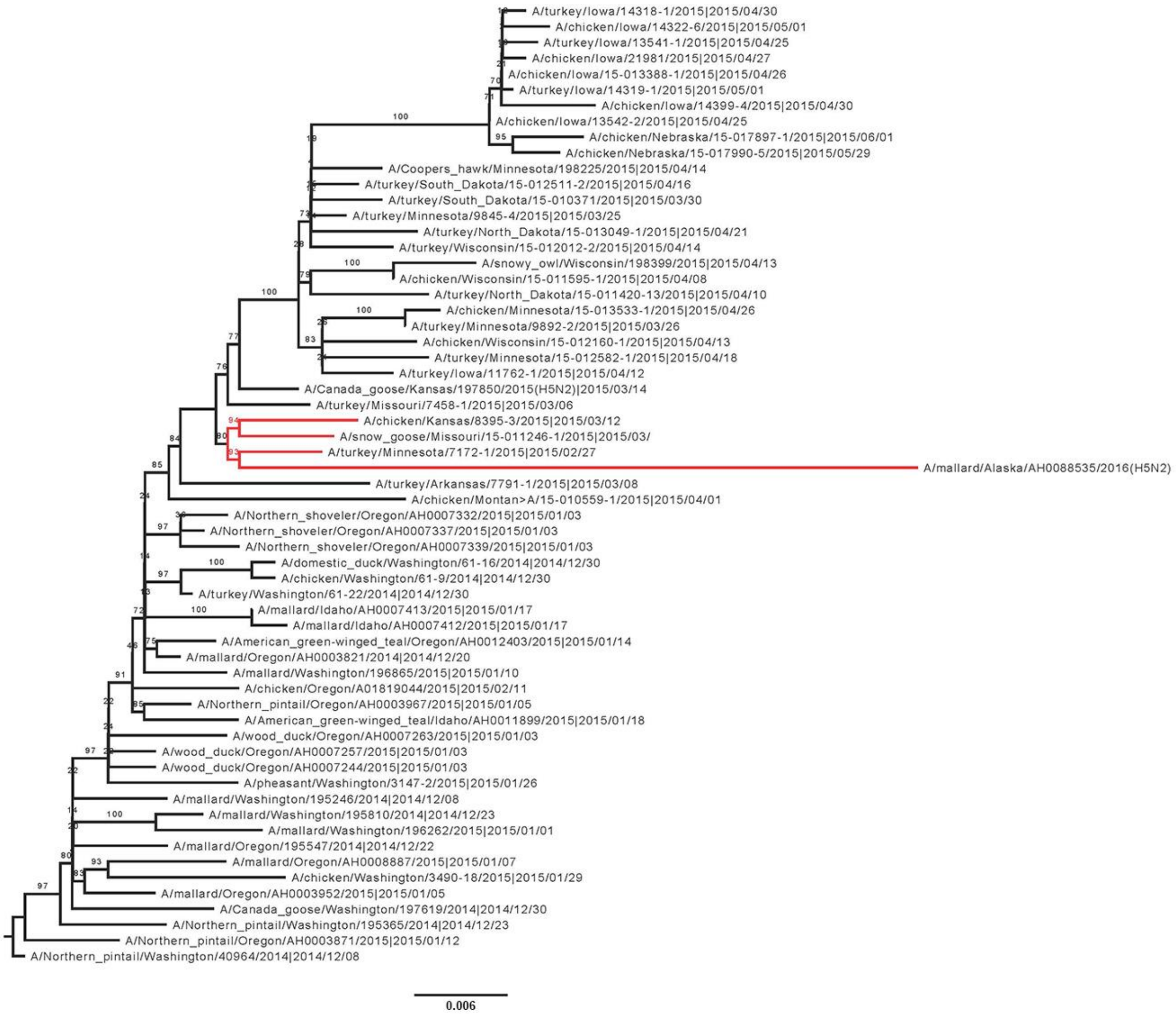

Technical Appendix Figure 1. Maximum-likelihood phylogeny of concatenated complete genome sequences of avian influenza A(H5N2) virus clade 2.3.4.4 in wild birds, Alaska, USA, 2016. Numbers along branches indicate bootstrap values >70%. Black circle indicates A/mallard/Alaska/AH0008887/2016(H5N2) virus. Red branches indicate a genetic cluster that includes the A/mallard/Alaska/AH0008887/2016(H5N2) virus and related viruses. Scale bar indicates nucleotide substitutions per site.

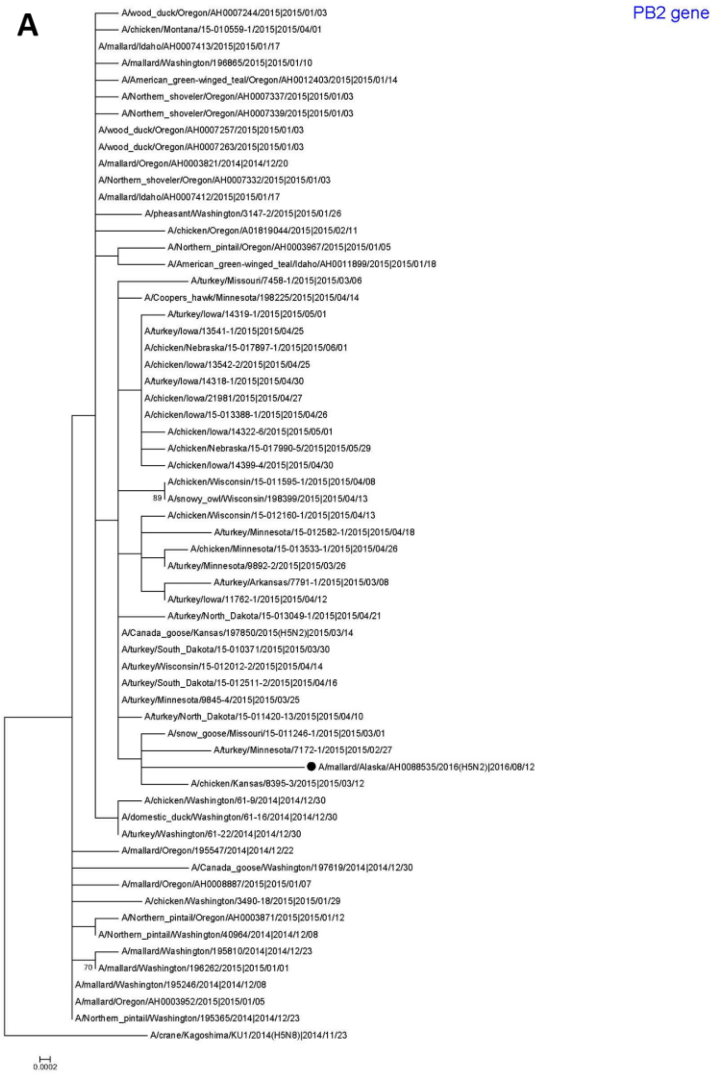
A
PB2 gene
A/wood_duck/Oregon/AH0007244/2015|2015/01/03
A/chicken/Montana/15-010559-1/2015|2015/04/01
A/mallard/Idaho/AH0007413/2015|2015/01/17
A/mallard/Washington/196865/2015|2015/01/10
A/American_green-winged_teal/Oregon/AH0012403/2015|2015/01/14
A/Northern_shoveler/Oregon/AH0007337/2015|2015/01/03
A/Northern_shoveler/Oregon/AH0007339/2015|2015/01/03
A/wood_duck/Oregon/AH0007257/2015|2015/01/03
A/wood_duck/Oregon/AH0007263/2015|2015/01/03
A/mallard/Oregon/AH0003821/2014|2014/12/20
A/Northern_shoveler/Oregon/AH0007332/2015|2015/01/03
A/mallard/Idaho/AH0007412/2015|2015/01/17
A/pheasant/Washington/3147-2/2015|2015/01/26
A/chicken/Oregon/A01819044/2015|2015/02/11
A/Northern_pintail/Oregon/AH0003967/2015|2015/01/05
A/American_green-winged_teal/Idaho/AH0011899/2015|2015/01/18
A/turkey/Missouri/7458-1/2015|2015/03/06
A/Coopers_hawk/Minnesota/198225/2015|2015/04/14
A/turkey/Iowa/14319-1/2015|2015/05/01
A/turkey/Iowa/13541-1/2015|2015/04/25
A/chicken/Nebraska/15-017897-1/2015|2015/06/01
A/chicken/Iowa/13542-2/2015|2015/04/25
A/turkey/Iowa/14318-1/2015|2015/04/30
A/chicken/Iowa/21981/2015|2015/04/27
A/chicken/Iowa/15-013388-1/2015|2015/04/26
A/chicken/Iowa/14322-6/2015|2015/05/01
A/chicken/Nebraska/15-017990-5/2015|2015/05/29
A/chicken/Iowa/14399-4/2015|2015/04/30
A/chicken/Wisconsin/15-011595-1/2015|2015/04/08
89
A/snowy_owl/Wisconsin/198399/2015|2015/04/13
A/chicken/Wisconsin/15-012160-1/2015|2015/04/13
A/turkey/Minnesota/15-012582-1/2015|2015/04/18
A/chicken/Minnesota/15-013533-1/2015|2015/04/26
A/turkey/Minnesota/9892-2/2015|2015/03/26
A/turkey/Arkansas/7791-1/2015|2015/03/08
A/turkey/Iowa/11762-1/2015|2015/04/12
A/turkey/North_Dakota/15-013049-1/2015|2015/04/21
A/Canada_goose/Kansas/197850/2015(H5N2)|2015/03/14
A/turkey/South_Dakota/15-010371/2015|2015/03/30
A/turkey/Wisconsin/15-012012-2/2015|2015/04/14
A/turkey/South_Dakota/15-012511-2/2015|2015/04/16
A/turkey/Minnesota/9845-4/2015|2015/03/25
A/turkey/North_Dakota/15-011420-13/2015|2015/04/10
A/snow_goose/Missouri/15-011246-1/2015|2015/03/01
A/turkey/Minnesota/7172-1/2015|2015/02/27
A/mallard/Alaska/AH0088535/2016(H5N2)|2016/08/12
A/chicken/Kansas/8395-3/2015|2015/03/12
A/chicken/Washington/61-9/2014|2014/12/30
A/domestic_duck/Washington/61-16/2014|2014/12/30
A/turkey/Washington/61-22/2014|2014/12/30
A/mallard/Oregon/195547/2014|2014/12/22
A/Canada_goose/Washington/197619/2014|2014/12/30
A/mallard/Oregon/AH0008887/2015|2015/01/07
A/chicken/Washington/3490-18/2015|2015/01/29
A/Northern_pintail/Oregon/AH0003871/2015|2015/01/12
A/Northern_pintail/Washington/40964/2014|2014/12/08
A/mallard/Washington/195810/2014|2014/12/23
70
A/mallard/Washington/196262/2015|2015/01/01
A/mallard/Washington/195246/2014|2014/12/08
A/mallard/Oregon/AH0003952/2015|2015/01/05
A/Northern_pintail/Washington/195365/2014|2014/12/23
A/crane/Kagoshima/KU1/2014(H5N8)|2014/11/23
0.0002

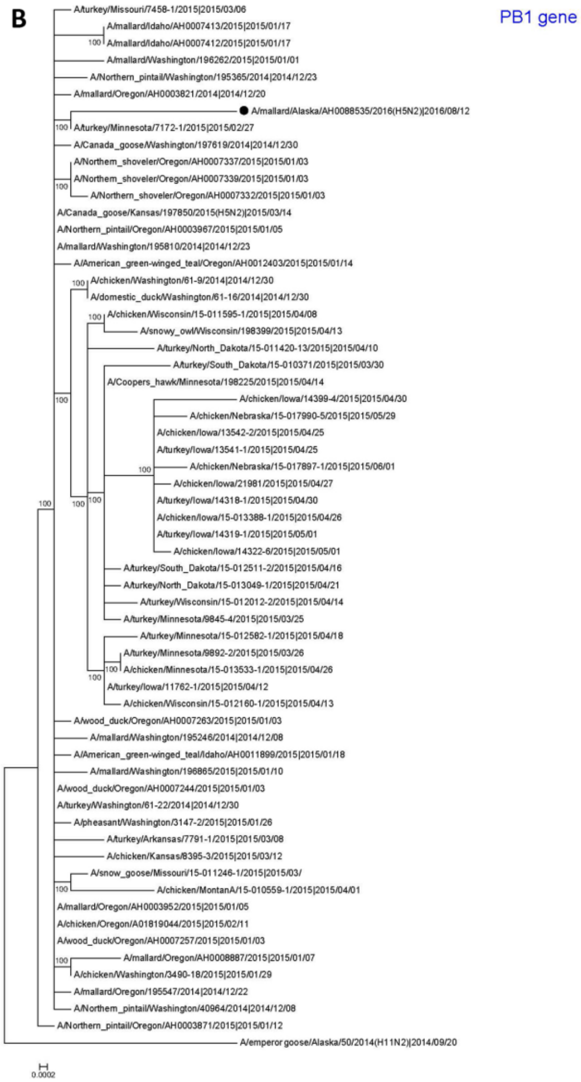
B
PB1 gene
A/turkey/Missouri/7458-1/2015|2015/03/06
A/mallard/Idaho/AH0007413/2015|2015/01/17
100
A/mallard/Idaho/AH0007412/2015|2015/01/17
A/mallard/Washington/196262/2015|2015/01/01
A/Northern_pintail/Washington/195365/2014|2014/12/23
A/mallard/Oregon/AH0003821/2014|2014/12/20
A/mallard/Alaska/AH0088535/2016(H5N2)|2016/08/12
100
A/turkey/Minnesota/7172-1/2015|2015/02/27
A/Canada_goose/Washington/197619/2014|2014/12/30
A/Northern_shoveler/Oregon/AH0007337/2015|2015/01/03
A/Northern_shoveler/Oregon/AH0007339/2015|2015/01/03
100
A/Northern_shoveler/Oregon/AH0007332/2015|2015/01/03
A/Canada_goose/Kansas/197850/2015(H5N2)|2015/03/14
A/Northern_pintail/Oregon/AH0003967/2015|2015/01/05
A/mallard/Washington/195810/2014|2014/12/23
A/American_green-winged_teal/Oregon/AH0012403/2015|2015/01/14
100
A/chicken/Washington/61-9/2014|2014/12/30
A/domestic_duck/Washington/61-16/2014|2014/12/30
100
A/chicken/Wisconsin/15-011595-1/2015|2015/04/08
A/snowy_owl/Wisconsin/198399/2015|2015/04/13
A/turkey/North_Dakota/15-011420-13/2015|2015/04/10
A/turkey/South_Dakota/15-010371/2015|2015/03/30
A/Coopers_hawk/Minnesota/198225/2015|2015/04/14
100
A/chicken/Iowa/14399-4/2015|2015/04/30
A/chicken/Nebraska/15-017990-5/2015|2015/05/29
A/chicken/Iowa/13542-2/2015|2015/04/25
A/turkey/Iowa/13541-1/2015|2015/04/25
100
A/chicken/Nebraska/15-017897-1/2015|2015/06/01
A/chicken/Iowa/21981/2015|2015/04/27
100
100
100
A/turkey/Iowa/14318-1/2015|2015/04/30
A/chicken/Iowa/15-013388-1/2015|2015/04/26
A/turkey/Iowa/14319-1/2015|2015/05/01
A/chicken/Iowa/14322-6/2015|2015/05/01
A/turkey/South_Dakota/15-012511-2/2015|2015/04/16
A/turkey/North_Dakota/15-013049-1/2015|2015/04/21
A/turkey/Wisconsin/15-012012-2/2015|2015/04/14
A/turkey/Minnesota/9845-4/2015|2015/03/25
A/turkey/Minnesota/15-012582-1/2015|2015/04/18
A/turkey/Minnesota/9892-2/2015|2015/03/26
100
A/chicken/Minnesota/15-013533-1/2015|2015/04/26
100
A/turkey/Iowa/11762-1/2015|2015/04/12
A/chicken/Wisconsin/15-012160-1/2015|2015/04/13
A/wood_duck/Oregon/AH0007263/2015|2015/01/03
A/mallard/Washington/195246/2014|2014/12/08
A/American_green-winged_teal/Idaho/AH0011899/2015|2015/01/18
A/mallard/Washington/196865/2015|2015/01/10
A/wood_duck/Oregon/AH0007244/2015|2015/01/03
A/turkey/Washington/61-22/2014|2014/12/30
A/pheasant/Washington/3147-2/2015|2015/01/26
A/turkey/Arkansas/7791-1/2015|2015/03/08
A/chicken/Kansas/8395-3/2015|2015/03/12
A/snow_goose/Missouri/15-011246-1/2015|2015/03/
100
A/chicken/MontanA/15-010559-1/2015|2015/04/01
A/mallard/Oregon/AH0003952/2015|2015/01/05
A/chicken/Oregon/A01819044/2015|2015/02/11
A/wood_duck/Oregon/AH0007257/2015|2015/01/03
100
A/mallard/Oregon/AH0008887/2015|2015/01/07
A/chicken/Washington/3490-18/2015|2015/01/29
A/mallard/Oregon/195547/2014|2014/12/22
A/Northern_pintail/Washington/40964/2014|2014/12/08
A/Northern_pintail/Oregon/AH0003871/2015|2015/01/12
A/emperor goose/Alaska/50/2014(H11N2)|2014/09/20
0.0002

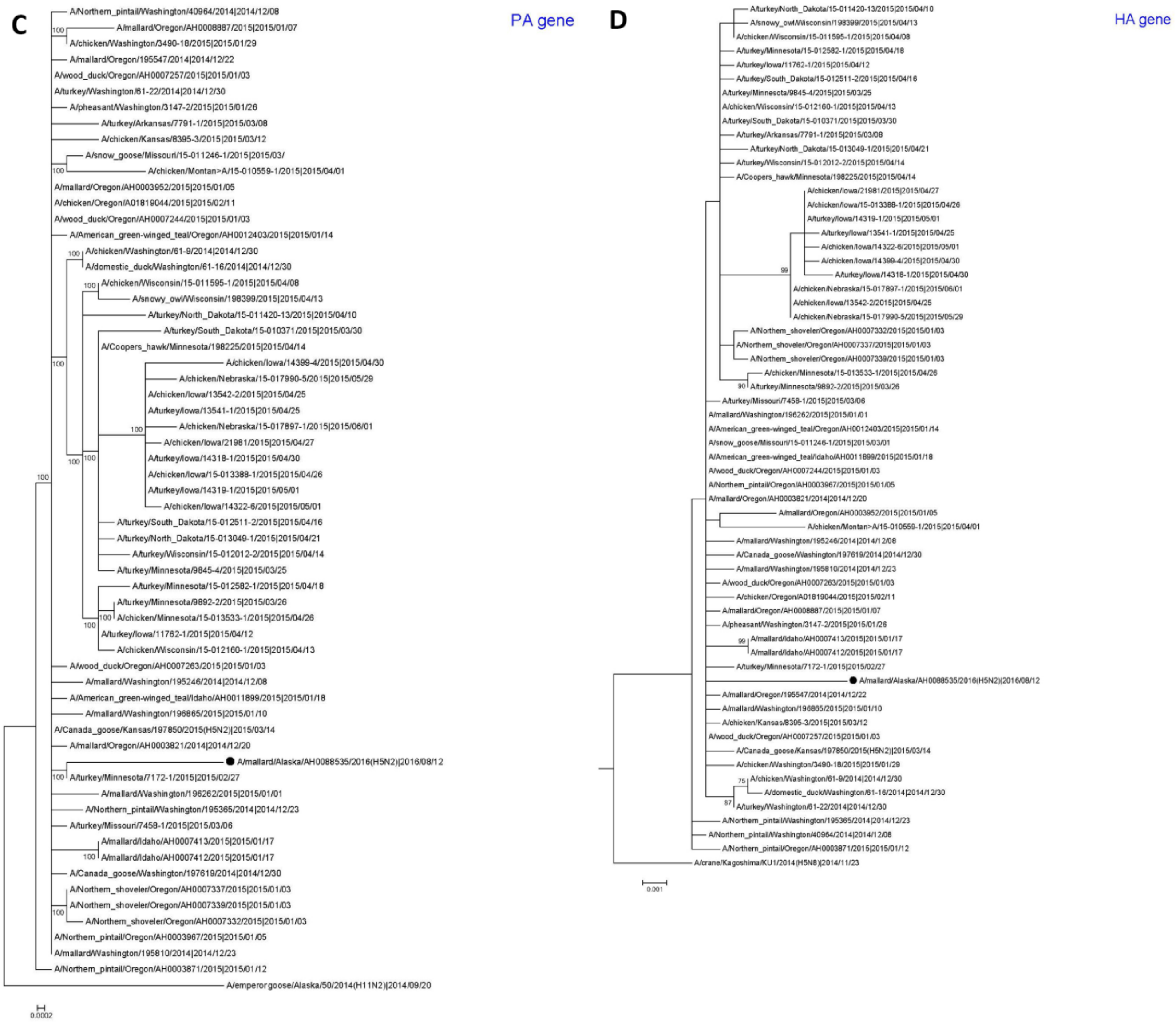

C
PA gene
A/Northern_pintail/Washington/40964/2014|2014/12/08
A/mallard/Oregon/AH0008887/2015|2015/01/07
A/chicken/Washington/3490-18/2015|2015/01/29
A/mallard/Oregon/195547/2014|2014/12/22
A/wood_duck/Oregon/AH0007257/2015|2015/01/03
A/turkey/Washington/61-22/2014|2014/12/30
A/pheasant/Washington/3147-2/2015|2015/01/26
A/turkey/Arkansas/7791-1/2015|2015/03/08
A/chicken/Kansas/8395-3/2015|2015/03/12
A/snow_goose/Missouri/15-011246-1/2015|2015/03/
A/chicken/Montan>A/15-010559-1/2015|2015/04/01
A/mallard/Oregon/AH0003952/2015|2015/01/05
A/chicken/Oregon/A01819044/2015|2015/02/11
A/wood_duck/Oregon/AH0007244/2015|2015/01/03
A/American_green-winged_teal/Oregon/AH0012403/2015|2015/01/14
A/chicken/Washington/61-9/2014|2014/12/30
A/domestic_duck/Washington/61-16/2014|2014/12/30
A/chicken/Wisconsin/15-011595-1/2015|2015/04/08
A/snowy_owl/Wisconsin/198399/2015|2015/04/13
A/turkey/North_Dakota/15-011420-13/2015|2015/04/10
A/turkey/South_Dakota/15-010371/2015|2015/03/30
A/Coopers_hawk/Minnesota/198225/2015|2015/04/14
A/chicken/Iowa/14399-4/2015|2015/04/30
A/chicken/Nebraska/15-017990-5/2015|2015/05/29
A/chicken/Iowa/13542-2/2015|2015/04/25
A/turkey/Iowa/13541-1/2015|2015/04/25
A/chicken/Nebraska/15-017897-1/2015|2015/06/01
A/chicken/Iowa/21981/2015|2015/04/27
A/turkey/Iowa/14318-1/2015|2015/04/30
A/chicken/Iowa/15-013388-1/2015|2015/04/26
A/turkey/Iowa/14319-1/2015|2015/05/01
A/chicken/Iowa/14322-6/2015|2015/05/01
A/turkey/South_Dakota/15-012511-2/2015|2015/04/16
A/turkey/North_Dakota/15-013049-1/2015|2015/04/21
A/turkey/Wisconsin/15-012012-2/2015|2015/04/14
A/turkey/Minnesota/9845-4/2015|2015/03/25
A/turkey/Minnesota/15-012582-1/2015|2015/04/18
A/turkey/Minnesota/9892-2/2015|2015/03/26
A/chicken/Minnesota/15-013533-1/2015|2015/04/26
A/turkey/Iowa/11762-1/2015|2015/04/12
A/chicken/Wisconsin/15-012160-1/2015|2015/04/13
A/wood_duck/Oregon/AH0007263/2015|2015/01/03
A/mallard/Washington/195246/2014|2014/12/08
A/American_green-winged_teal/Idaho/AH0011899/2015|2015/01/18
A/mallard/Washington/196865/2015|2015/01/10
A/Canada_goose/Kansas/197850/2015(H5N2)|2015/03/14
A/mallard/Oregon/AH0003821/2014|2014/12/20
A/mallard/Alaska/AH0088535/2016(H5N2)|2016/08/12
A/turkey/Minnesota/7172-1/2015|2015/02/27
A/mallard/Washington/196262/2015|2015/01/01
A/Northern_pintail/Washington/195365/2014|2014/12/23
A/turkey/Missouri/7458-1/2015|2015/03/06
A/mallard/Idaho/AH0007413/2015|2015/01/17
A/mallard/Idaho/AH0007412/2015|2015/01/17
A/Canada_goose/Washington/197619/2014|2014/12/30
A/Northern_shoveler/Oregon/AH0007337/2015|2015/01/03
A/Northern_shoveler/Oregon/AH0007339/2015|2015/01/03
A/Northern_shoveler/Oregon/AH0007332/2015|2015/01/03
A/Northern_pintail/Oregon/AH0003967/2015|2015/01/05
A/mallard/Washington/195810/2014|2014/12/23
A/Northern_pintail/Oregon/AH0003871/2015|2015/01/12
A/emperor goose/Alaska/50/2014(H11N2)|2014/09/20
0.0002
D
HA gene
A/turkey/North_Dakota/15-011420-13/2015|2015/04/10
A/snowy_owl/Wisconsin/198399/2015|2015/04/13
A/chicken/Wisconsin/15-011595-1/2015|2015/04/08
A/turkey/Minnesota/15-012582-1/2015|2015/04/18
A/turkey/Iowa/11762-1/2015|2015/04/12
A/turkey/South_Dakota/15-012511-2/2015|2015/04/16
A/turkey/Minnesota/9845-4/2015|2015/03/25
A/chicken/Wisconsin/15-012160-1/2015|2015/04/13
A/turkey/South_Dakota/15-010371/2015|2015/03/30
A/turkey/Arkansas/7791-1/2015|2015/03/08
A/turkey/North_Dakota/15-013049-1/2015|2015/04/21
A/turkey/Wisconsin/15-012012-2/2015|2015/04/14
A/Coopers_hawk/Minnesota/198225/2015|2015/04/14
A/chicken/Iowa/21981/2015|2015/04/27
A/chicken/Iowa/15-013388-1/2015|2015/04/26
A/turkey/Iowa/14319-1/2015|2015/05/01
A/turkey/Iowa/13541-1/2015|2015/04/25
A/chicken/Iowa/14322-6/2015|2015/05/01
A/chicken/Iowa/14399-4/2015|2015/04/30
A/turkey/Iowa/14318-1/2015|2015/04/30
A/chicken/Nebraska/15-017897-1/2015|2015/06/01
A/chicken/Iowa/13542-2/2015|2015/04/25
A/chicken/Nebraska/15-017990-5/2015|2015/05/29
A/Northern_shoveler/Oregon/AH0007332/2015|2015/01/03
A/Northern_shoveler/Oregon/AH0007337/2015|2015/01/03
A/Northern_shoveler/Oregon/AH0007339/2015|2015/01/03
A/chicken/Minnesota/15-013533-1/2015|2015/04/26
A/turkey/Minnesota/9892-2/2015|2015/03/26
A/turkey/Missouri/7458-1/2015|2015/03/06
A/mallard/Washington/196262/2015|2015/01/01
A/American_green-winged_teal/Oregon/AH0012403/2015|2015/01/14
A/snow_goose/Missouri/15-011246-1/2015|2015/03/01
A/American_green-winged_teal/Idaho/AH0011899/2015|2015/01/18
A/wood_duck/Oregon/AH0007244/2015|2015/01/03
A/Northern_pintail/Oregon/AH0003967/2015|2015/01/05
A/mallard/Oregon/AH0003821/2014|2014/12/20
A/mallard/Oregon/AH0003952/2015|2015/01/05
A/chicken/Montan>A/15-010559-1/2015|2015/04/01
A/mallard/Washington/195246/2014|2014/12/08
A/Canada_goose/Washington/197619/2014|2014/12/30
A/mallard/Washington/195810/2014|2014/12/23
A/wood_duck/Oregon/AH0007263/2015|2015/01/03
A/chicken/Oregon/A01819044/2015|2015/02/11
A/mallard/Oregon/AH0008887/2015|2015/01/07
A/pheasant/Washington/3147-2/2015|2015/01/26
A/mallard/Idaho/AH0007413/2015|2015/01/17
A/mallard/Idaho/AH0007412/2015|2015/01/17
A/turkey/Minnesota/7172-1/2015|2015/02/27
A/mallard/Alaska/AH0088535/2016(H5N2)|2016/08/12
A/mallard/Oregon/195547/2014|2014/12/22
A/mallard/Washington/196865/2015|2015/01/10
A/chicken/Kansas/8395-3/2015|2015/03/12
A/wood_duck/Oregon/AH0007257/2015|2015/01/03
A/Canada_goose/Kansas/197850/2015(H5N2)|2015/03/14
A/chicken/Washington/3490-18/2015|2015/01/29
A/chicken/Washington/61-9/2014|2014/12/30
A/domestic_duck/Washington/61-16/2014|2014/12/30
A/turkey/Washington/61-22/2014|2014/12/30
A/Northern_pintail/Washington/195365/2014|2014/12/23
A/Northern_pintail/Washington/40964/2014|2014/12/08
A/Northern_pintail/Oregon/AH0003871/2015|2015/01/12
A/crane/Kagoshima/KU1/2014(H5N8)|2014/11/23
0.001

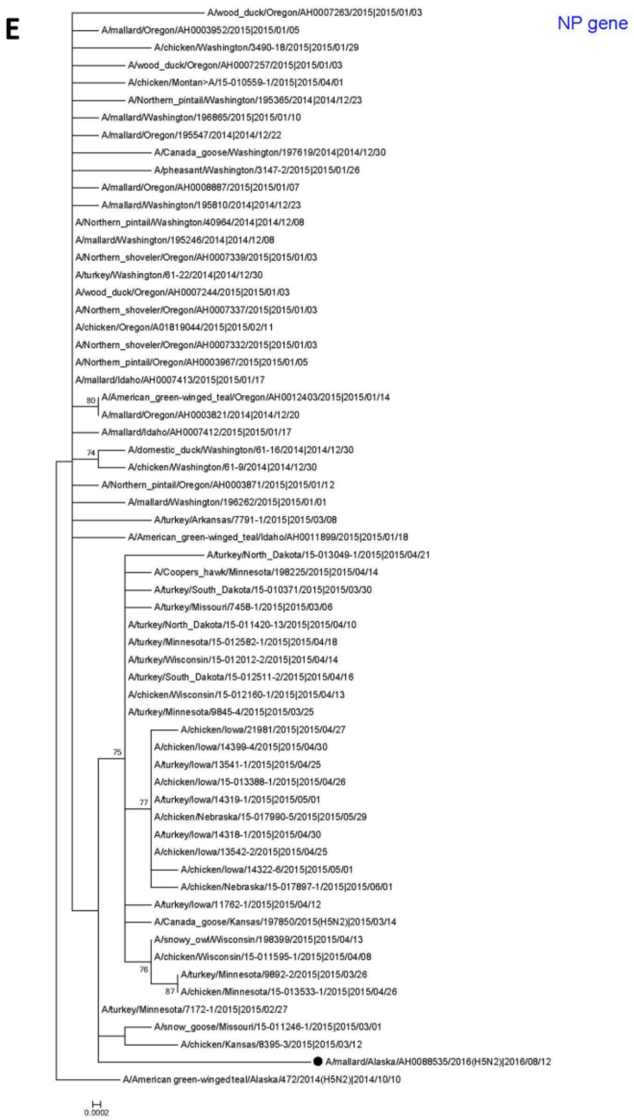
E
NP gene

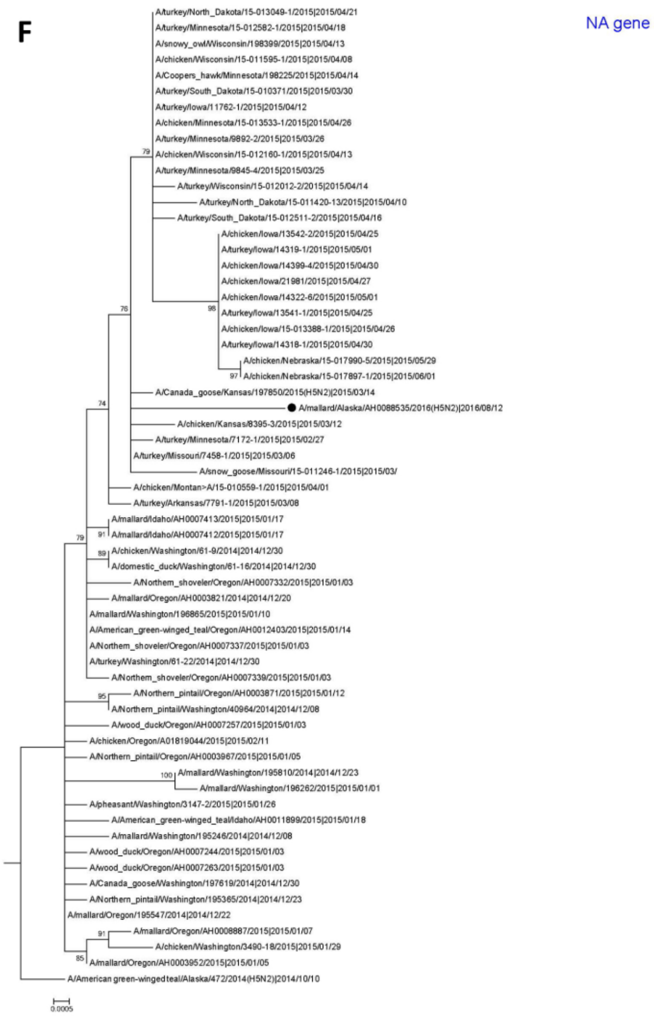
F
NA gene

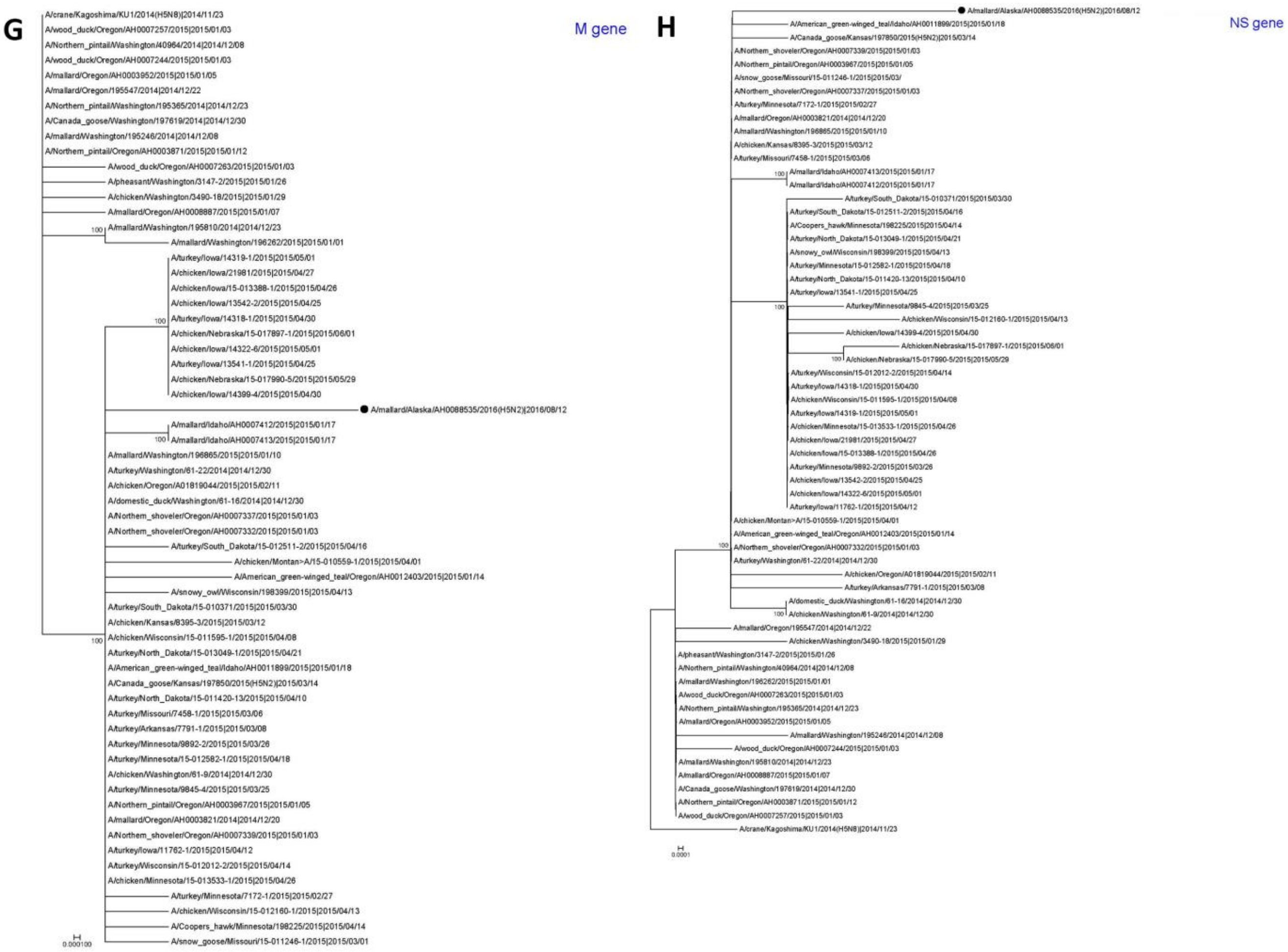

Technical Appendix Figure 2. Maximum-likelihood phylogeny of A) polymerase basic 2 (PB2); B) polymerase basic 1 (PB1); C) polymerase acidic (PA); D) hemagglutinin (HA); E) nucleoprotein (NP); F) neuraminidase (NA); G) matrix (M), and H) nonstructural (NS) protein genes of avian influenza A(H5N2) virus clade 2.3.4.4 in wild birds, Alaska, USA, 2016. Numbers along branches indicate bootstrap values >70%. Black circle indicates A/mallard/Alaska/AH0008887/2016(H5N2) virus. Scale bars indicate nucleotide substitutions per site.

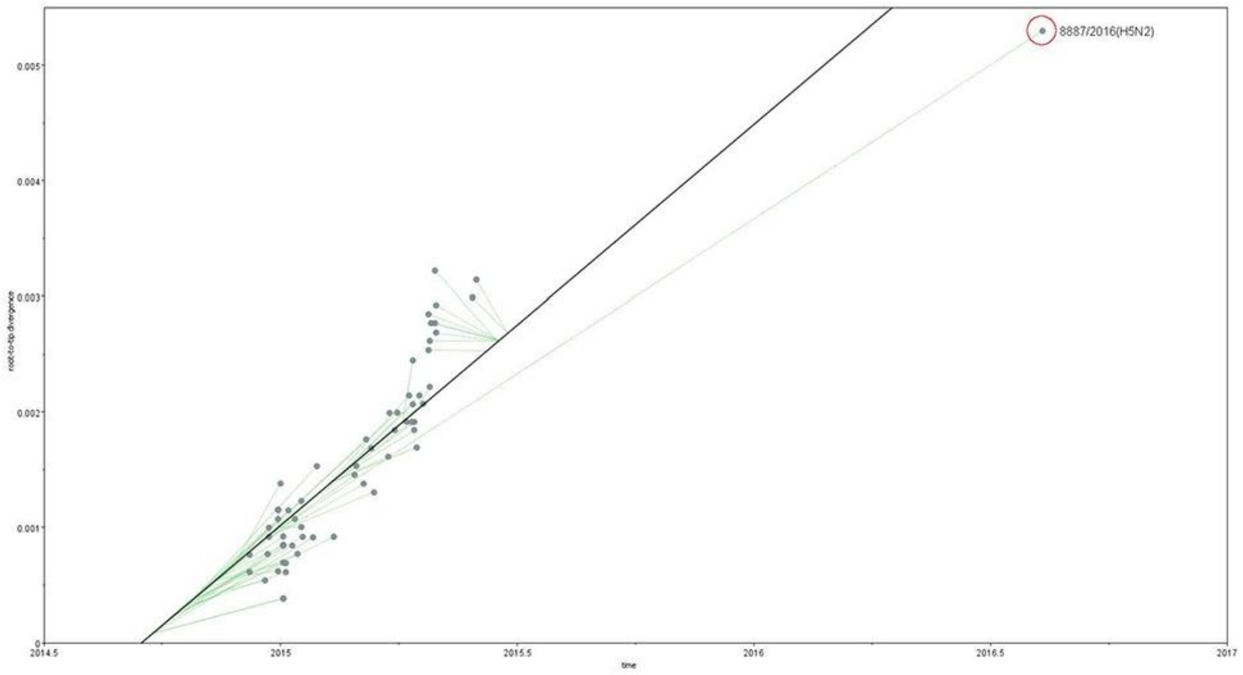

Technical Appendix Figure 3. Root-to-tip regression plot, with ancestor traces shown, of maximum-likelihood phylogeny of concatenated complete genome sequences of avian influenza A(H5N2) virus clade 2.3.4.4 in wild birds, Alaska, USA, 2016. Red circle indicates A/mallard/Alaska/AH0008887/2016(H5N2) virus.
